# Exposure to a Pathological Condition May Be Required for the Cells to Secrete Exosomes Containing mtDNA Aberration

**DOI:** 10.1155/2022/7960198

**Published:** 2022-03-17

**Authors:** Manjusha Vaidya, Sandeep Sreerama, Mariana Gaviria, Kiminobu Sugaya

**Affiliations:** Burnett School of Biomedical Sciences, College of Medicine, University of Central Florida, Florida, USA

## Abstract

Exosomes, nanovesicles secreted by all cells, carry out intercellular communication by transmitting biologically active cargo comprising DNA, RNA, and proteins. These biomolecules reflect the status of their parent cells and can be altered by pathological conditions. Therefore, the researchers have been investigating differential sequences and quantities of DNA associated with exosomes as valuable biomarkers of diseases. Exosomes carry different types of DNA molecules, including genomic, cytoplasmic, and mitochondrial (mtDNA). The mtDNA aberrations are reported to be a hallmark of diseases involving oxidative stress, such as cancer and neurodegenerative diseases. Establishing robust *in vitro* models comprising appropriate cell lineages is the first step towards investigating disease-specific anomalies and testing therapeutics. Induced pluripotent stem (iPS) cells from patients with diseases have been used for this purpose since they can differentiate into various cells. The current study investigated mtDNA aberrations in exosomes secreted by primary cancer cells and neural stem cells (NSCs) differentiated from iPS cells. The primary cancer cells were isolated from surgically removed glioblastoma multiforme (GBM) tissue, and the iPS cells were produced from control and Alzheimer's disease (AD) subjects' B lymphocytes. We detected aberrations in mtDNA associated with exosomes secreted from GBM cells but not from the NSCs. This result indicates that the cells may not secrete exosomes carrying mtDNA aberration without exposure to a pathological condition. Thus, we may need to consider this fact when we use iPS cell-derived cells as an *in vitro* disease model.

## 1. Introduction

Exosomes are nanovesicles (30-130 nm) of late endocytic origin. Almost all types of cells secrete them for paracrine and endocrine intercellular communication (1). They can cross the blood-brain barrier (BBB) and anatomical compartments by transcytosis to be detected in peripheral blood and other body fluids (2). The exosome-associated DNA is found to carry disease-specific mutations as well as differential sequences resulting from disease progression and response to treatment. Mitochondrial DNA (mtDNA) in exosomes is a functionally active cargo. Its horizontal transfer to a new cell mediates hormonal therapy resistance in metastatic breast cancer, according to the research by Sansone et al. (3). Preexisting mutations of maternally inherited mtDNA are known to have clinical implications. On the other hand, it is well established that mtDNA is prone to prolific damages such as mutation and degradation due to the lack of a robust repair mechanism, absence of histone-like protective proteins, and exposure to oxidants. Thus, the pathological cellular environment of a diseased cell is also known to cause changes in the mtDNA sequence (4, 5). Mitochondria are the ATP-generating powerhouses of a cell that utilize an oxidative phosphorylation system (OXPHOS). The crucial component proteins of OXPHOS subunits, except for subunit II, are encoded by genes in mtDNA. Therefore, mutations and differential sequences in mtDNA can be severely detrimental to cell function and survival (6). Impaired mitochondrial function and mutations in mtDNA are associated with many neurodegenerative diseases like Alzheimer's disease (AD) and cancers, including glioblastoma multiforme (GBM) (7–9). mtDNA alterations are useful as diagnostic and prognostic markers in liquid biopsy for GBM management (10). The mtDNA carrying mutations is transported to adjacent cells via different modes, potentially creating a niche for the disease in a healthy cell, thereby contributing to disease pathology (11). Although mitochondria move to neighbor cells via tunneling nanotubes, mtDNA is known to migrate from one cell to another as an exosomal cargo. The population of mitochondria, the amount of DNA per mitochondrion, and the rate of DNA mutations vary depending on the disease. For example, mitochondria of various cancer cells possess more DNA coding for genes responsible for bioenergetics. Increased mtDNA copy numbers are reported in GBM differentiating cells and are associated with the initiation and maintenance of tumorigenesis (12–14). Antibiotic- or cancer-treatment-induced genotoxic shock may potentially send mtDNA to the exosome surface (13, 15). Radiation exposure is reported to elevate the mtDNA levels in exosomes secreted by human fibroblast cells (16). It is also reported that in the early stages of AD, the frequency of mtDNA mutations is remarkably high (7). These instances indicate that qualitative and quantitative changes in mtDNA are associated with the disease, its specific stage, progression, and response to therapy. Therefore, disease-specific modulations in exosome-associated mtDNA can serve as prognostic, diagnostic, and therapeutic intervention biomarkers. Analysis of mutations, single nucleotide polymorphisms (SNP), and deletions in the exosomal mtDNA secreted by cell lines cultured in vitro is an essential first step towards establishing their clinical validity.

## 2. Materials and Methods

### 2.1. Cell Culture

Human neural stem cells (NSC) were procured from Lonza (# PT2599). GBM cells were obtained from the resected mass of a tumor newly diagnosed via magnetic resonance imaging. The patient had not undergone GBM treatment earlier and had provided the Institutional Review Board-approved informed consent for the research study before the surgery. HIPPA regulations were strictly followed. A detailed characterization of these cell lines has been described in our previously published research (17). The tumor cells were dissociated for *in vitro* culture under the human subject protection protocol approved by AdventHealth and the University of Central Florida Institutional Review Board. The cell lines were grown in suspension cultures in growth media comprising heparin 5000 U (0.5 U/ml), EGF 20 ng/ml, bFGF 20 ng/ml, and 2% B27 stock mixed in DMEM/F12. This suspension culture media does not require fetal bovine serum. Therefore, there is no interference of bovine exosomes. The induced pluripotent stem (iPS) cells derived from AD (iPS-AD) cell line were purchased from Coriell cell repositories (# CW50018). This cell line is reprogrammed from B lymphocytes of a subject diagnosed with AD. The diagnosis is based on the presence of A*β* peptide in the cerebrospinal fluid as well as affirmative ApoE4 carrier status (18). As a control for iPS-AD cells, iPS cells reprogrammed from B lymphocytes from a subject with no cognitive decline (iPS-control) were acquired from the Coriell cell repository (# CW50064). Following the protocol by Pauly et al., the iPS-control and iPS-AD cells were cultured to make iPS-derived neural stem cells (iPS-NSC and iPS-NSC-AD, respectively) (19). The conditioned media was collected for the isolation of exosomes after validation of neural stem cells by immunostaining and gene expression studies using SOX2, NESTIN, PAX6, and MSI1.

### 2.2. Exosome Isolation

The conditioned media of the cell cultures was centrifuged at 10,000 × *g* for 30 minutes to remove cell remnants. The supernatant was transferred to a new tube, and 5 ml of 20% PEG and 200 *μ*l of 7.5 M NaCl were added to each 10 ml volume to precipitate the exosomes (20). Upon overnight incubation at 4°C, the supernatant was centrifuged at 10,000 × *g* for 60 minutes. The exosome pellet was resuspended in 1x PBS (pH 7.4, without calcium and magnesium). The manufacturer's protocol was followed to purify the exosomes further using CD63 antibody-conjugated magnetic beads (Thermo Fisher Scientific: Invitrogen Exosome Human CD63 Isolation/Detection Reagent (from cell culture media), Ref #10606D).

### 2.3. PCR Settings and Electrophoresis

Because this is a qualitative analysis, no exosomal DNA was extracted or quantified. Instead, a 15 *μ*l exosomal suspension was used directly in place of the template for 50 *μ*l PCR reaction volume. Using High-Performance GoTaq® G2 DNA Polymerase (Promega) and mtDNA specific primer pairs (Table 1), the PCR reactions were set up as follows: 94°C—5 minutes (denaturation: 94°C—30 seconds, annealing: 50°C—30 seconds, extension: 72°C—2 minutes) × 30, 72°C—10 minutes. The PCR products were run on 1.5% agarose gel in 1x TAE buffer.

### 2.4. Cloning of PCR Products in pCR4TOPO-TA Vector

After gel electrophoresis of the PCR products, the DNA samples were eluted using QIAquick® Gel Extraction Kit (Qiagen, catalog # 28706) as per their protocol. Following the manufacturer's protocol, the PCR products were ligated with the pCR4TOPO-TA vector (Invitrogen™ TOPO™ TA Cloning™ Kit for Sequencing, catalog # 450030), transformed into chemically competent E. coli (*Stbl3*) cells, and selected on LB agar with ampicillin (100 *μ*g/ml). Upon overnight incubation at 37°C, the colonies were picked, grown in LB with ampicillin, and the plasmid DNA was extracted using QIAprep Spin Miniprep Kit (QIAGEN #27104). On average, five clones for each PCR product-sequencing vector ligation were sent for Sanger sequencing with GENEWIZ®. With four cell lines and four primer pairs amplifying the mtDNA of a cell line, altogether, eighty clones were sent. The BLAST analysis of the clones is provided in the supplementary section along with the four-color chromatograms showing the results of the respective sequencing runs Figures S-1–S-3.

### 2.5. Exosomal DNA Sequence Analysis

To eliminate the pCR4TOPO-TA vector sequences that could skew the analysis and the results, the nucleotide sequences received from GENEWIZ® were plugged into VacScreen from NCBI. Simultaneously, the forward and reverse primer sequences were identified, and the exact PCR product sequence was analyzed using the Basic Local Alignment Search Tool (BLAST). Standard BLAST search and human BLAST search were performed to investigate the identities of the PCR product. Exosomal mtDNA was also checked using the mtDNA-specific tool MITOMAP (https://http://www.mitomap.org/mitomaster/index.cgi). SNP found in the sequences was scrutinized for clinical significance using the NCBI SNP database.

### 2.6. Statistical Analysis

An ordinary two-way ANOVA was performed on the mean SNP number of each cell line per primer pair, followed by Tukey's multiple comparison test that compares individual differences. Software GraphPad Prism 9.3.0 was used to perform the analyses.

## 3. Results

The current study is aimed at investigating whether the iPS cell-based *in vitro* disease model can reproduce the exosomal mtDNA aberrations resulting from a cellular environment changed by the disease. We hypothesized that cells exposed to the disease would secrete exosomes with variations in mtDNA sequence because of the stress generated by the disease pathology. Therefore, exosomal mtDNA secreted by iPS-AD derived from peripheral tissue, which has never been exposed to a pathological condition, such as oxidative stress, may not have any variations in the mtDNA sequence. As the diseased models, we used primary cells from GBM tissue and the NSCs derived from iPS-AD. As the controls, we used fetal NSCs and NSCs derived from iPS cells of healthy subjects. We analyzed the specific regions of exosomal mtDNA, reported to show differential sequences in these diseases (3, 21–23) for the clonal variations of nucleotides and SNPs. This approach may help amplify and analyze the specific region of mtDNA sequence in the exosome because it may be randomly fragmented and complex to sequence the entire mitochondria genome by next-generation sequencing. Also, identifying new molecular markers for these diseases is not the purpose of this study.

**Figure 1:**
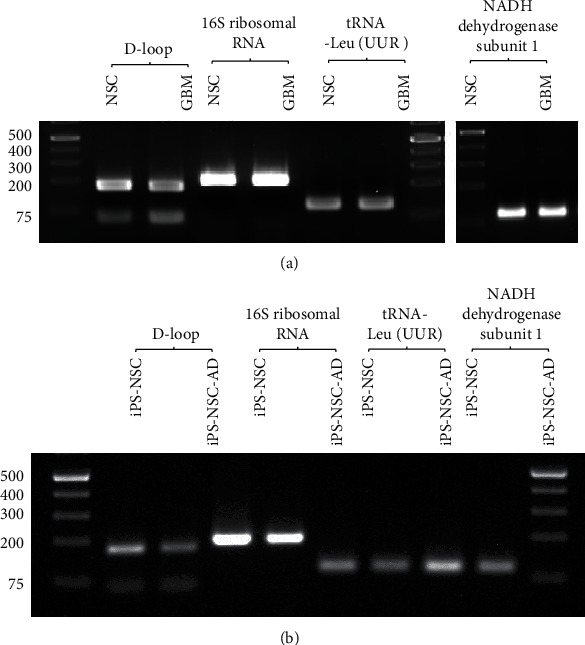
Typical gel images of exosomal mtDNA PCR products using primer sets described in Table 1. (a) NSC and GBM in the figure represent the mtDNA PCR products using exosomes isolated from neural stem cells (NSC) and glioblastoma multiforme (GBM) cell culture media as the sample. (b) iPS-NSC and iPS-NSC-AD in the figure represent the mtDNA PCR products using exosomes isolated from NSCs derived from iPS cells reprogrammed from B lymphocytes of control (iPS-NSC) and AD (iPS-NSC-AD) subjects.

We used primer sets to amplify the specific region of mtDNA, which are reported to be associated with these diseases, namely, D-loop, 16S ribosomal RNA, tRNA-Leu-UUR, and NADH dehydrogenase subunit 1, which are described in Table 1.  Figure 1 shows a typical gel electrophoresis image of amplified exosomal mtDNA.

**Figure 2:**
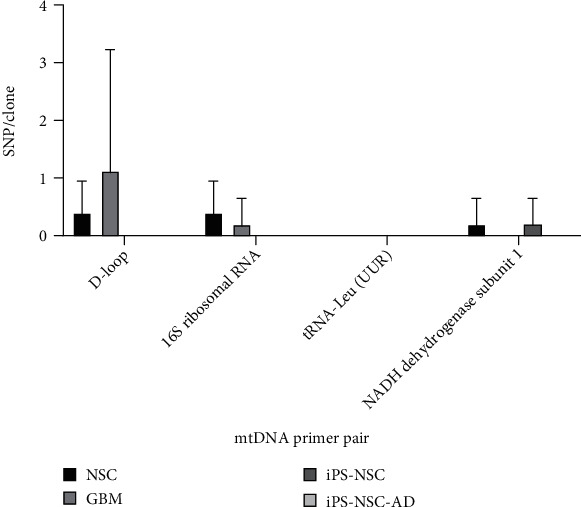
The number of the SNPs in clones obtained from the primer pair sets for D-loop, 16S ribosomal RNA, tRNA-Leu (UUR), and NADH dehydrogenase subunit 1 of mtDNA associated with exosomes released by neural stem cells (NSC), glioblastoma multiforme (GBM), control subject iPS-derived NSC (iPS-NSC), and AD subject iPS cells (iPS-NSC-AD). The numbers of SNPs in each group (*n* = 5) are presented as mean ± SD. Two-way ANOVA followed by Tukey's multiple comparison *post hoc* analysis was performed, and there was no significant difference among the groups (*P* > 0.05).

In two-way ANOVA, the SNP analysis of each clone for all the cell types tested and primer pair showed a slight variation with no statistical significance (Figure 2). However, in-depth BLAST analysis of the clone obtained with each primer pair revealed SNPs in GBM cells, underscoring the heteroplasmy in exosomal mtDNA. The BLAST analysis of the clones and the SNPs and their MITOMAP reports are discussed in detail in the following section.

### 3.1. Displacement Loop (D-loop) 321-496

**Figure 3:**
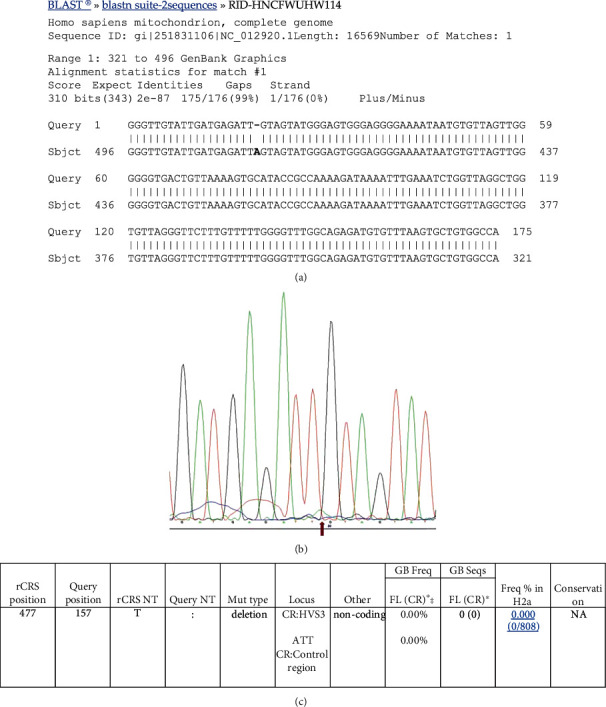
NSC exosome D-loop clone. (a) BLAST analysis against *Homo sapiens* mitochondrion, complete genome NC_012920.1. CD63+ exosomal mtDNA amplified with D-loop primers (location: 321-496). PCR products were cloned into the pCR4-TOPO-TA vector. (b) The red arrow in the sequence figure indicates the position of the nucleotide deletion in exosomal DNA in *Homo sapiens* mitochondrion complete genome sequence (NC_012920.1). The entire chromatogram of the clone is provided in the supplementary section (Figure S-7). (c) Singular SNP at 477 (A>-) found in the clone (bold letter in (a)) is also reported in MITOMAP at rCRS position 477 (T>-). Information from MITOMAP: ^∗^the current GB frequency data is derived from two sets of human mitochondrial sequences from GenBank: 52,633 full-length (FL) sequences (>15.4 kbp) and 74,970 short, control region (CR) containing sequences (0.4-1.6 kbp); ^‡^high-frequency haplogroups: variants found in haplogroups at 50% or higher are marked with a flag.

BLAST analysis revealed that PCR primers amplified the expected regions of mtDNA associated with GBM and iPS-NSC-AD exosomes and the mtDNA of their normal counterparts. While NSC exosomes had barely one SNP per clone in two of the clones (*n* = 2) (Figure 3), BLAST analysis has identified over 9 SNP together in two clones of GBM exosomal mtDNA (*n* = 9). Where four-color chromatograms show the results of the sequencing run, the red arrows point to the SNPs (Supplementary Figures S-4 and S-5). These SNPs are listed in Table 2. Where some of these SNPs are reported in the MITOMAP database, others are novel. This finding supports the research that the D-loop region is prime for point mutations and SNP with pathogenic relevance to GBM (5, 24). Out of three highly polymorphic hypervariable segments of D-loop (HVI: 16024–16383, HVII: 57–372, and HVIII: 438–574), we have partially amplified regions II and III (321-496). Although most SNP is also reported in MITOMAP, their presence in GBM exosomal DNA is significant. Not only in gliomas and other types of cancers, D-loop mutations and SNP are also reported in AD (7). For the amplified D-loop region 321-496, we did not find any SNP or mutations in the iPS-NSC-AD exosomes (Supplementary Figure S-6). An effect of aging, nucleotide variations accumulate in mtDNA, including the highly susceptible D-loop region, and the donor's age for iPSC-AD B-lymphocytes used in our study was 58 years, old enough to have possible nucleotide differences in the mtDNA sequence (25). Additionally, mutation load is reported to be significantly higher in neuronal mitochondria in AD (7). Although the B lymphocyte donor was diagnosed with the disease, the mtDNA in exosomes did not reflect the mutations.

### 3.2. 16S Ribosomal RNA (16S mt-rRNA) 1873-2078

The clones analyzed for regions 1873-2078 have shown very few nucleotide variations irrespective of the cell source and disease. GBM exosomal PCR products showed a novel deletion at 1884 C>-, which has not been reported in MITOMAP. Two different clones of NSC exosomal DNA had two SNPs per clone. Where SNP at 1893 (A>-) is novel, SNP at 1900 T>C is reported in MITOMAP (Table 2). Elson et al. have reported several nucleotide variations and mutations in 16S mt rRNA that can disrupt the activity of the small ribosomal subunit and have implications in various cancers (26). We did not find any of these SNP in the region amplified.

### 3.3. tRNA-Leu (UUR) 3212-3319

In the tRNA-Leu (UUR) region, a transition type of pathogenic mutation found in mt-tRNA-Leu (UUR) at 3243 A>G is most frequent in humans. It is associated with mitochondrial encephalopathy, lactic acidosis, stroke-like episodes, and diabetes (27, 28). It is also reported that the tRNA-Leu (UUR) region is a prime spot for pathogenic mtDNA mutations (29). In our study, neither GBM/NSC nor iPSC-NSC/iPSC-NSC-AD showed any SNP in the exosomal mtDNA for region 3212-3319 of tRNA-Leu (UUR).

### 3.4. NADH Dehydrogenase Subunit 1 (ND1) 3458-3561

Where NSC-derived exosomes had one SNP at 3502 T>A (also reported in MITOMAP), GBM PCR products matched 100% with the human mitochondrial genome (NC_012920.1). Similarly, iPS-NSC had single and novel SNP at 3545 (C>-) not reported in MITOMAP (Table 2). In MITOMAP, an SNP is reported at 3546 (C>-) with a frameshift. Mutations in ND1 are implicated in colorectal carcinoma, hepatocellular carcinoma, and human gastric cancer, and SNP in this mtDNA gene is studied as a potential risk factor for breast cancer (30–33). In addition, mutations in this mitochondrial gene have prognostic value in postoperative renal cell carcinoma (34).

## 4. Discussion

Unlike nuclear DNA, mtDNA undergoes fewer epigenetic changes. For example, methylation of the D-loop region is crucial in neurodegenerative diseases and cancers alike. Although methylation levels of mtDNA are significantly lower than nuclear DNA, this type of regulation has a potential role in replicating mtDNA and transcription of genes. Mutations in D-loop can compromise its methylation status (35). In a clustered data analysis, GBM clones do not show significant variation in the mean value. However, when the nucleotide variants are present in a clone, their frequency is higher in the D-loop region, known for elevated susceptibility to mutations. Yeung et al. have also checked the D-loop in cultured NSC to see the effect of culture conditions on nucleotide variation in this region. They detected two variations, the same amount found in NSC exosomal mtDNA, although at different nucleotides in the sequence (9). This underscores the importance of the D-loop region's differential sequences found in the exosomes of GBM. The same research group has also reported that some mtDNA-coding regions such as ND1 are unaffected in GBM. They found no variants in this gene. Our GBM exosomal mtDNA clones do not have any SNP either. This finding agrees with their data suggesting that the lack of mtDNA variation at the source reflects the SNP-devoid status in exosomal mtDNA.

Like GBM, neuronally extracted mtDNA with Alzheimer's pathology has shown significantly elevated mutations in the D-loop (7). Hoekstra et al. have demonstrated that the mutation load in this noncoding D-loop region is many folds higher than that in the coding region. This ratio is independent of the AD condition. However, in our study, the iPSC-NSC-AD exosomes had 100% identity to the reported sequence and no SNP. Although the donor was diagnosed with AD as described before, the iPSCs were created using B lymphocytes, which lacked AD pathology found in neural cells and mitochondria. In particular, the sporadic change in mtDNA is highly dependent on the environment. Thus, the iPS cell-derived exosomal mtDNA may have failed to reflect the sequence and nucleotide variations attributed to degeneration or pathological changes in neural cells. This indicates that although the researchers have successfully established iPS-derived neuronal models to study AD as well as other neurodegenerative diseases and established mtDNA as one of the disease biomarkers, iPS model-derived exosomal mtDNA may not be ideal for the biomarker studies (36, 37).

In addition to the primers described in Table 1, other regions of exosomal mtDNA were amplified by PCR. However, the PCR for NADH, DHG-sub2, ATP-6, MTCYB, 12S ribosomal RNA, and an 8842-nucleotide long fragment (position 5999-14841) did not yield any product. However, all the primer sets successfully amplified the target genes when cytoplasmic DNAs were used as templates, indicating that mtDNAs associated with exosomes are fragmented. Fragmented mtDNAs are reported to be released from defective mitochondria during their turnover and hauled away for clearance by exosomes and other extracellular vesicles (38).

## 5. Conclusions

Only the exosomal mtDNA from GBM cells showed aberrations as reported by other researchers in these specific regions, while the exosomal mtDNA from fetal NSCs and iPS-derived NSCs were almost identical to the sequences reported in GenBank. The GBM cells are derived from a resected tumor tissue, and they are directly exposed to the disease environment. The iPS-NSC-AD cells, which are reprogrammed from B lymphocytes, are not subjected to AD pathology, although it has been differentiated in NSCs. Thus, exposure to the pathological environments may be needed for the cells to secrete exosomes that carry mtDNA aberrations. The cell culture model based on the specific cell linage derived from iPS cells, which has been used to investigate many diseases, may fall short of reproducing the disease-related intracellular environments, which reflect the exosomal mtDNA. In order to avoid this issue, we may need to use primary neural cells directly from the patient's brain to investigate the effect of AD pathology in some cases. Another note is that we did not detect the difference in exosomal mtDNA sequence secreted by primary NSC, iPS-NSC-AD, and iPS-NSC cells. This may indicate that reprograming process to produce iPS cells may not affect mtDNA sequence.

## Figures and Tables

**Table 1:** PCR primers and their sequences used for exosomal mtDNA amplification.

Mitochondrial region	Location on NC_012920.1	Size (bp)	Primer pair [forward (F)/reverse (R)]	Reference
D-loop	321-496	175	F: 5′-TGGCCACAGCACTTAAACACATCTC-3′	(3)
			R: 5′-GGGTTGTATTGATGAGATTAGTAGTATGGGAG-3′	
16S ribosomal RNA	1873-2078	205	F: 5′-AACTTTGCAAGGAGAGCCAAAGC-3′	(3)
			R: 5′-GGGATTTAGAGGGTTCTGTGGGC-3′	
tRNA-Leu-UUR	3212-3319	107	F: 5′-CACCCAAGAACAGGGTTTGT-3′	(21, 22)
			R: 5′-TGGCCATGGGTATGTTGTTA-3′	
NADH dehydrogenase subunit 1	3458-3561	103	F: 5′-ACGCCATAAAACTCTTCACCAAAG-3′	(23)
			R: 5′-TAGTAGAAGAGCGATGGTGAGAGCTA-3′	

**Table 2:** A concise account of the SNP found in exosomal mtDNA. D-loop region 321-496 in GBM has a significantly higher occurrence of SNP over other regions and cell lines. Except for one SNP found at position 3545 (C>-) in NADH dehydrogenase subunit 1 region 3458-3561, the rest of the iPS-NSC and iPS-NSC-AD cell line clones had 100% identity to the reported sequences in the human mitochondrial genome (NC_012920.1) for all other genes. Each SNP in a clone is unique irrespective of the primer pair or a cell line.

Exosomal cell source	mtDNA region	Position on NC_012920.1	SNP
NSC	D-loop	321-496	477 A>-, 334 T>-
GBM	D-loop	321-496	371 G>N, 426 T>A, 420 G>N, 404 G>T, 481: G>N, 480: A>T, 478: T>N, 477: A>N and 379: T>C, 480: T>A
NSC	16S ribosomal RNA	1873-2078	1893 A>-, 1900 T>C
GBM	16S ribosomal RNA	1873-2078	1884 C>-
GBM	tRNA-Leu-UUR	3212-3319	None
NSC	tRNA-Leu-UUR	3212-3319	None
NSC	NADH dehydrogenase subunit 1	3458-3561	3502, T>A
iPS-NSC	NADH dehydrogenase subunit 1	3458-3561	3545 C>-

## Data Availability

No data were used to support this study.

## Supplementary Materials

Supplementary materials Figure S-1: human BLAST analysis of the exosomal mtDNA clones. PCR product was obtained using 16S ribosomal RNA primers that amplify region 1873-2078 (Homo sapiens mitochondrion, complete genome NC_012920.1). The amplification product was ligated with Invitrogen™ pCR4TOPO-TA sequencing vector. The clones were selected on ampicillin and Sanger sequenced by GENEWIZ®. The sequences are analyzed using the Basic Local Alignment Search Tool (BLAST). SNPs are highlighted by a red rectangle. (A) NSC exosomes: SNP @ 1893 A>-. (B) GBM exosomes: deletion/SNP @ 1884 C>-. (C) iPS-NSC and iPS-NSC-AD have 100% sequence identity with each other as well as with mitochondrial genomic sequence and no SNP. Figure S-2: human BLAST analysis of the exosomal mtDNA clones. PCR product obtained using tRNA-Leu (UUR) primers that amplify region 3212-3319 (Homo sapiens mitochondrion, complete genome NC_012920.1). The amplification product was ligated with Invitrogen™ pCR4TOPO-TA sequencing vector. The clones were selected on ampicillin and Sanger sequenced by GENEWIZ®. The sequences are analyzed using the Basic Local Alignment Search Tool (BLAST). NSC, GBM, iPS-NSC, and iPS-NSC-AD exosomal clones share 100% identity with each other as well as with mitochondrial genomic sequence. Figure S-3: human BLAST analysis of the exosomal mtDNA clones. PCR product obtained using NADH dehydrogenase subunit 1 primers that amplify region 3458-3561 (Homo sapiens mitochondrion, complete genome NC_012920.1). The amplification product was ligated with Invitrogen™ pCR4TOPO-TA sequencing vector. The clones were selected on ampicillin and Sanger sequenced by GENEWIZ®. The sequences are analyzed using the Basic Local Alignment Search Tool (BLAST). SNPs are highlighted by a red rectangle. (A) NSC exosomes: SNP @ 3502, T>A. (B) iPS-NSC exosomes: SNP @ 3545 C>-. (C) GBM and iPS-NSC-AD exosomes have 100% sequence identity with each other as well as with mitochondrial genomic sequence and no SNP. Figure S-4: GBM exosome D-loop clone 1. (A) BLAST analysis against Homo sapiens mitochondrion, complete genome NC_012920.1: CD63+ exosomal mtDNA was amplified with D-loop primers (location: 321-496). PCR products were cloned into the pCR4-TOPO-TA vector. SNPs at 371 G>N, 426 T>A, 420 G>N, and 404 G>T. (B) rCRS position according to MITOMAP: at 404: C>A, at 426: A>T, mutation type transversion. Information from MITOMAP: ^∗^the current GB frequency data is derived from two sets of human mitochondrial sequences from GenBank: 52,633 full-length (FL) sequences (>15.4 kbp) and 74,970 short, control region (CR) containing sequences (0.4-1.6 kbp); ^‡^high-frequency haplogroups: variants found in haplogroups at 50% or higher are marked with a flag. (C) The chromatogram depicts the sequence of the entire PCR product cloned. The red arrows correspond to the SNPs. Figure S-5: GBM exosome D-loop clone 2. (A) BLAST analysis against Homo sapiens mitochondrion, complete genome NC_012920.1. CD63+ exosomal mtDNA was amplified with D-loop primers (location: 321-496). PCR products were cloned into the pCR4-TOPO-TA vector. SNPs at 481: G>N, 480: A>T, 478: T>N, 477: A>N, and 379: T>C. (B) rCRS positions according to MITOMAP: @379: A>G (mutation type: transition), at 480: T>A, (mutation type transversion). Information from MITOMAP: ^∗^the current GB frequency data is derived from two sets of human mitochondrial sequences from GenBank: 52,633 full-length (FL) sequences (>15.4 kbp) and 74,970 short, control region (CR) containing sequences (0.4-1.6 kbp); ^‡^high-frequency haplogroups: variants found in haplogroups at 50% or higher are marked with a flag. (C) Because the clone has identical sequence to the one in Figure S-4, except for the SNPs, the chromatogram shows only the unique SNPs here, marked by a red arrow. Figure S-6: (A) iPS-NSC exosomes and (B) iPS-NSC-AD exosomes, D-loop clones. BLAST analysis against Homo sapiens mitochondrion, complete genome NC_012920.1. CD63+ exosomal mtDNA was amplified with D-loop primers (location: 321-496). PCR products were cloned into the pCR4-TOPO-TA vector. With 100% identity to the reported sequences, there are no SNPs in this region. (C) The chromatogram depicts the sequence of the entire PCR product cloned. Figure S-7: human BLAST analysis of the exosomal mtDNA NSC exosome D-loop clone. A detailed chromatogram for Figure 3(b). PCR product was obtained using D-loop RNA primers that amplify region 321-496 (Homo sapiens mitochondrion, complete genome NC_012920.1). The amplification product was ligated with Invitrogen™ pCR4TOPO-TA sequencing vector. The clones were selected on ampicillin and Sanger sequenced by GENEWIZ®. The sequences are analyzed using the Basic Local Alignment Search Tool (BLAST). The red arrow in the sequence figure indicates the position of the nucleotide deletion in exosomal DNA in Homo sapiens mitochondrion complete genome sequence (NC_012920.1).
