# Supplementary material for: Exposure to a Pathological Condition May Be Required for the Cells to Secrete Exosomes Containing mtDNA Aberration
Source: J Nucleic Acids. 2022 Mar 17;2022:7960198. doi: 10.1155/2022/7960198 (PMC9020996; doi:10.1155/2022/7960198)
Supplement: Supplementary materials — Figure S-1: human BLAST analysis of the exosomal mtDNA clones. PCR product was obtained using 16S ribosomal RNA primers that amplify region 1873-2078 (Homo sapiens mitochondrion, complete genome NC_012920.1). The amplification product was ligated with Invitrogen™ pCR4TOPO-TA sequencing vector. The clones were selected on ampicillin and Sanger sequenced by GENEWIZ®. The sequences are analyzed using the Basic Local Alignment Search Tool (BLAST). SNPs are highlighted by a red rectangle. (A) NSC exosomes: SNP @ 1893 A>-. (B) GBM exosomes: deletion/SNP @ 1884 C>-. (C) iPS-NSC and iPS-NSC-AD have 100% sequence identity with each other as well as with mitochondrial genomic sequence and no SNP. Figure S-2: human BLAST analysis of the exosomal mtDNA clones. PCR product obtained using tRNA-Leu (UUR) primers that amplify region 3212-3319 (Homo sapiens mitochondrion, complete genome NC_012920.1). The amplification product was ligated with Invitrogen™ pCR4TOPO-TA sequencing vector. The clones were selected on ampicillin and Sanger sequenced by GENEWIZ®. The sequences are analyzed using the Basic Local Alignment Search Tool (BLAST). NSC, GBM, iPS-NSC, and iPS-NSC-AD exosomal clones share 100% identity with each other as well as with mitochondrial genomic sequence. Figure S-3: human BLAST analysis of the exosomal mtDNA clones. PCR product obtained using NADH dehydrogenase subunit 1 primers that amplify region 3458-3561 (Homo sapiens mitochondrion, complete genome NC_012920.1). The amplification product was ligated with Invitrogen™ pCR4TOPO-TA sequencing vector. The clones were selected on ampicillin and Sanger sequenced by GENEWIZ®. The sequences are analyzed using the Basic Local Alignment Search Tool (BLAST). SNPs are highlighted by a red rectangle. (A) NSC exosomes: SNP @ 3502, T>A. (B) iPS-NSC exosomes: SNP @ 3545 C>-. (C) GBM and iPS-NSC-AD exosomes have 100% sequence identity with each other as well as with mitochondrial genomic sequence and no SNP. Figure S-4: GBM exosome D [file 7960198.f1.zip › S-1_rev2.docx]

Supplementary material: **Figure S-1**

**[A]**


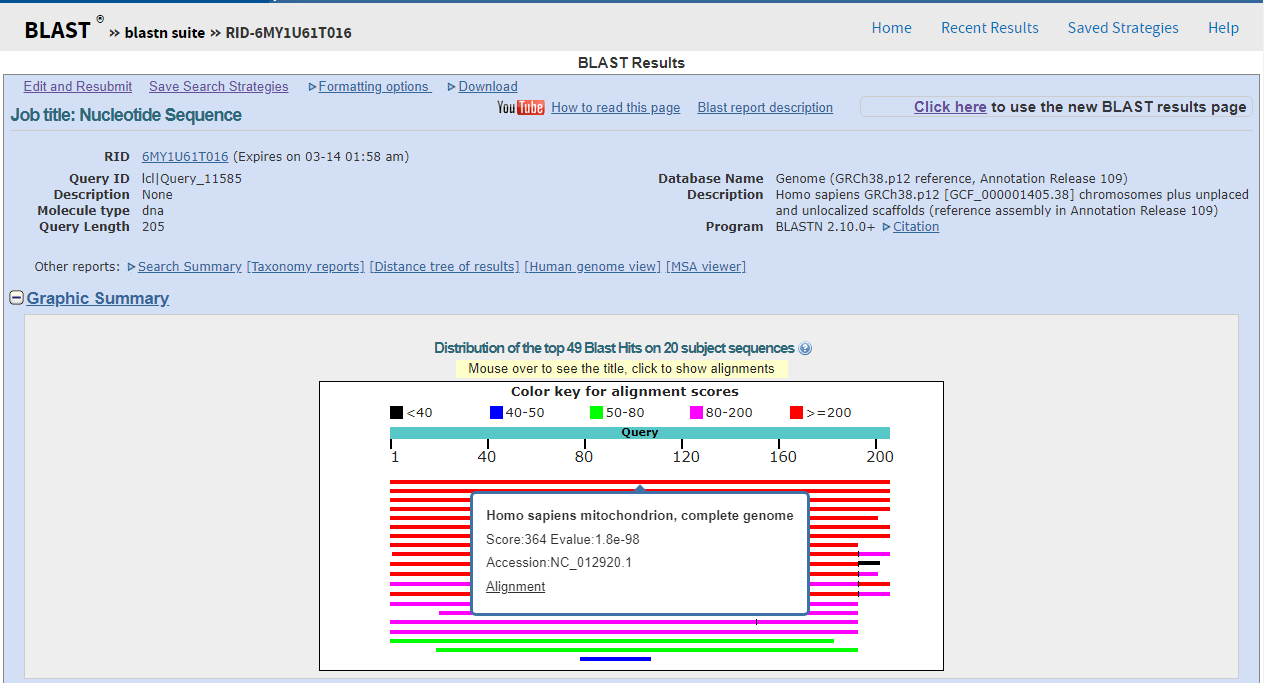

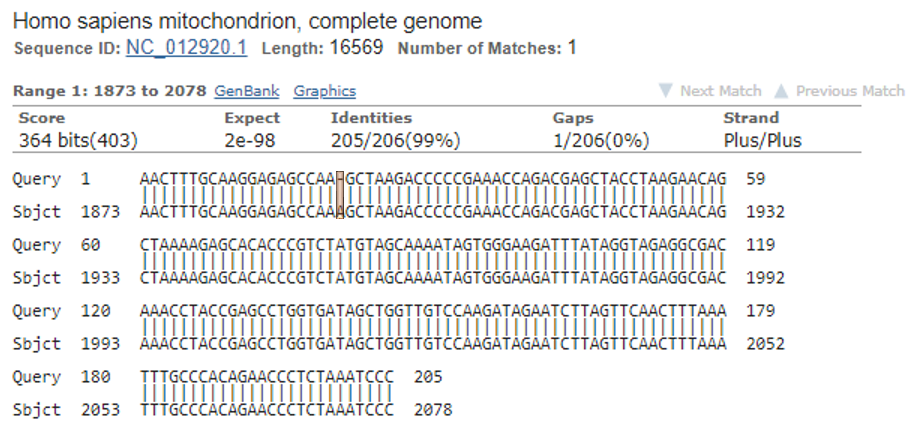


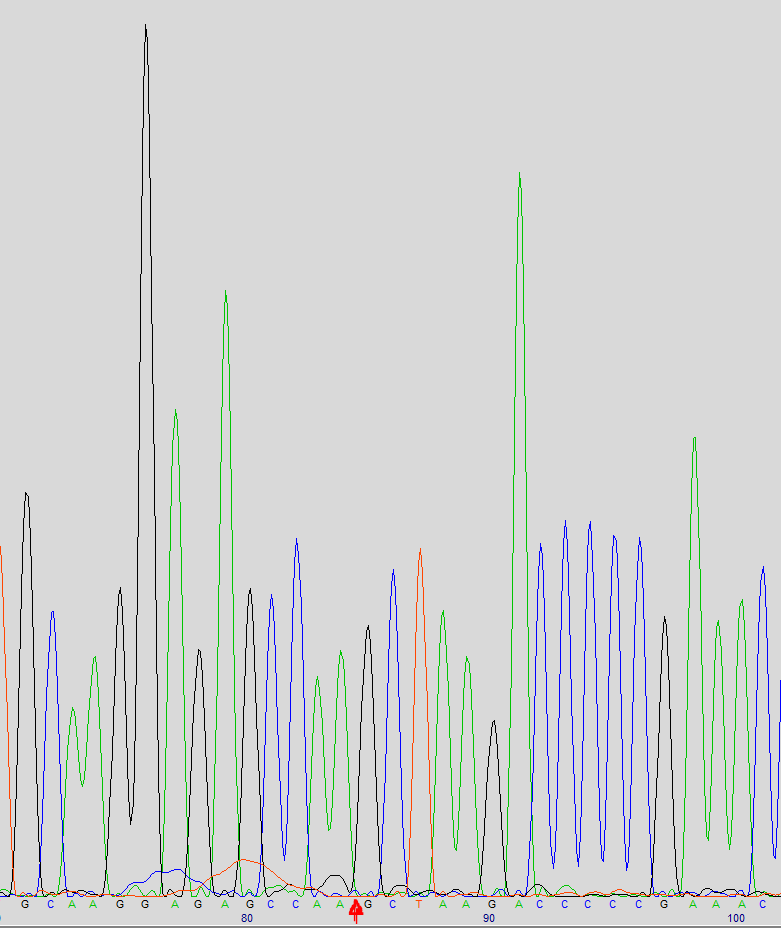


**[B]**


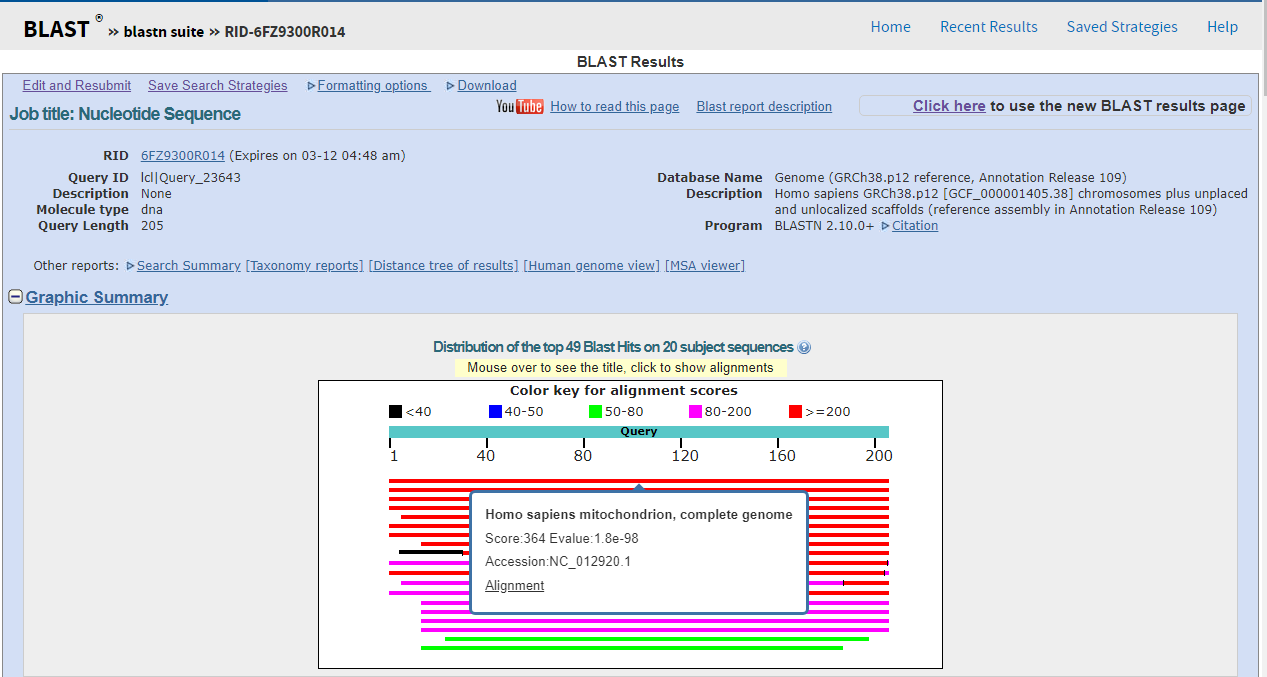


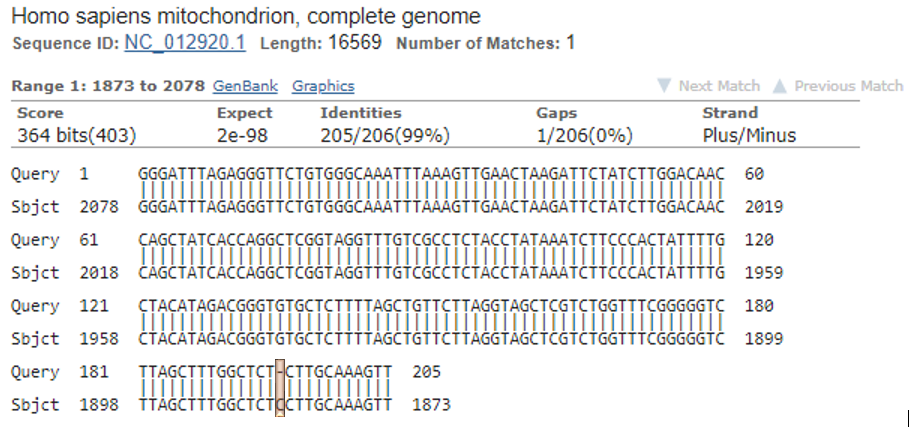


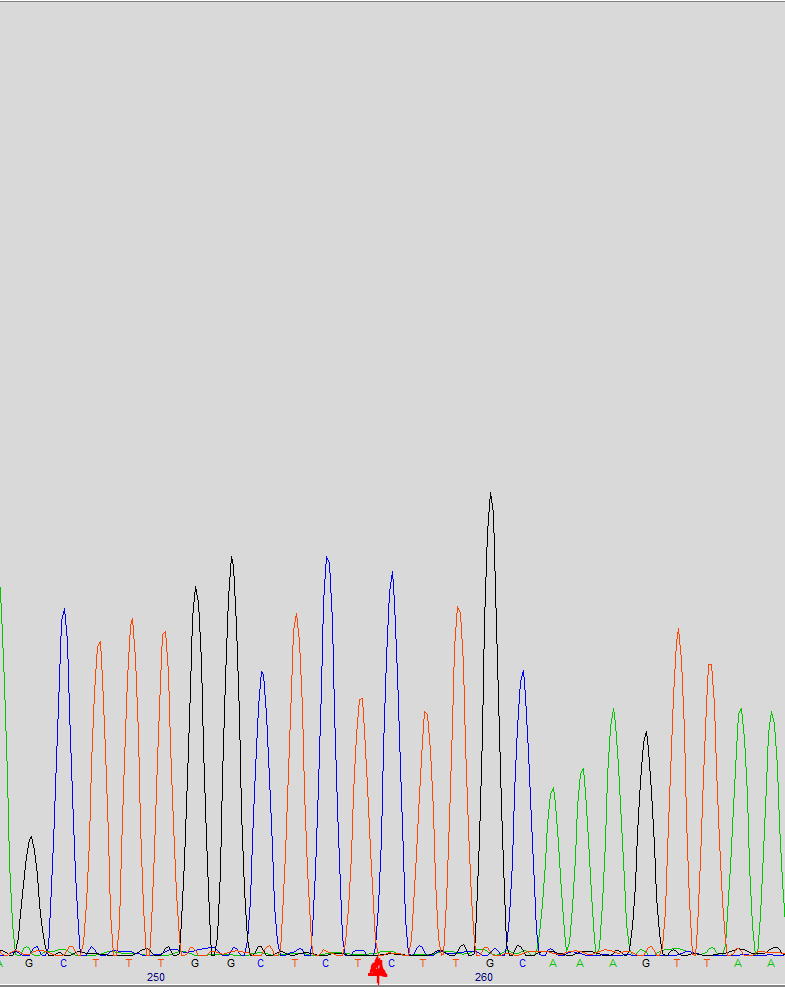


**[C]**


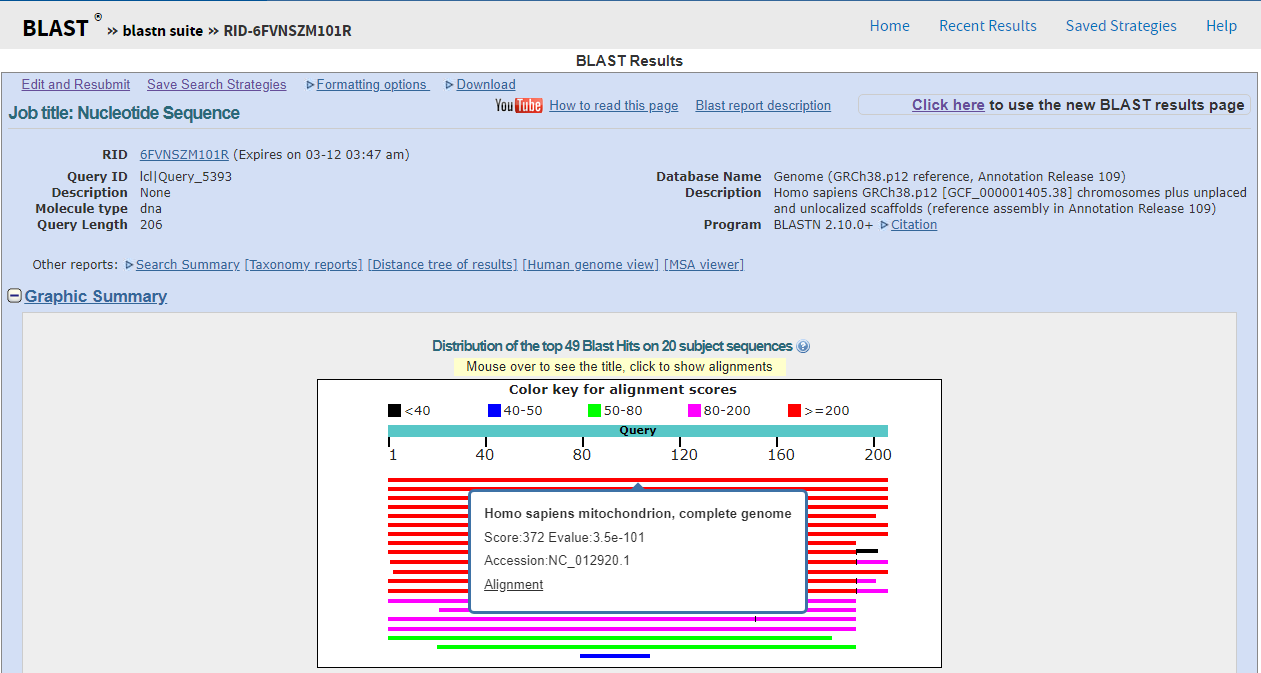


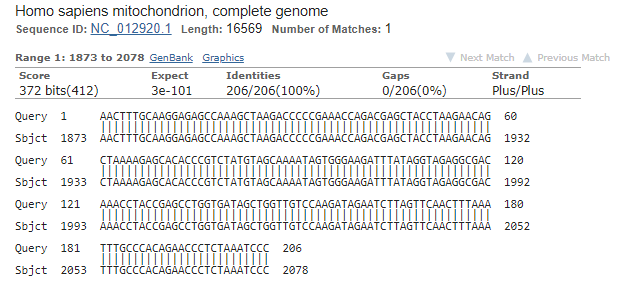


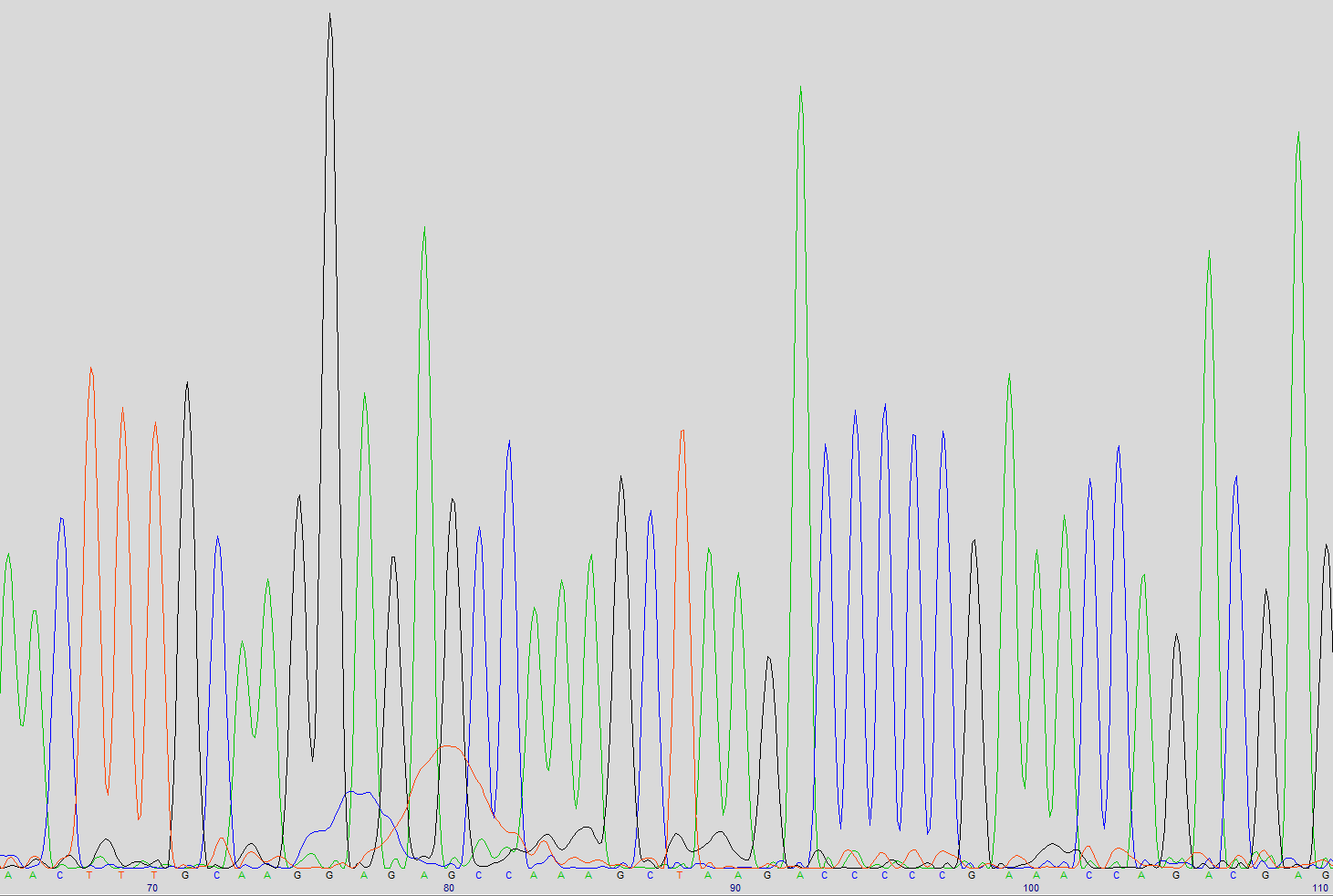


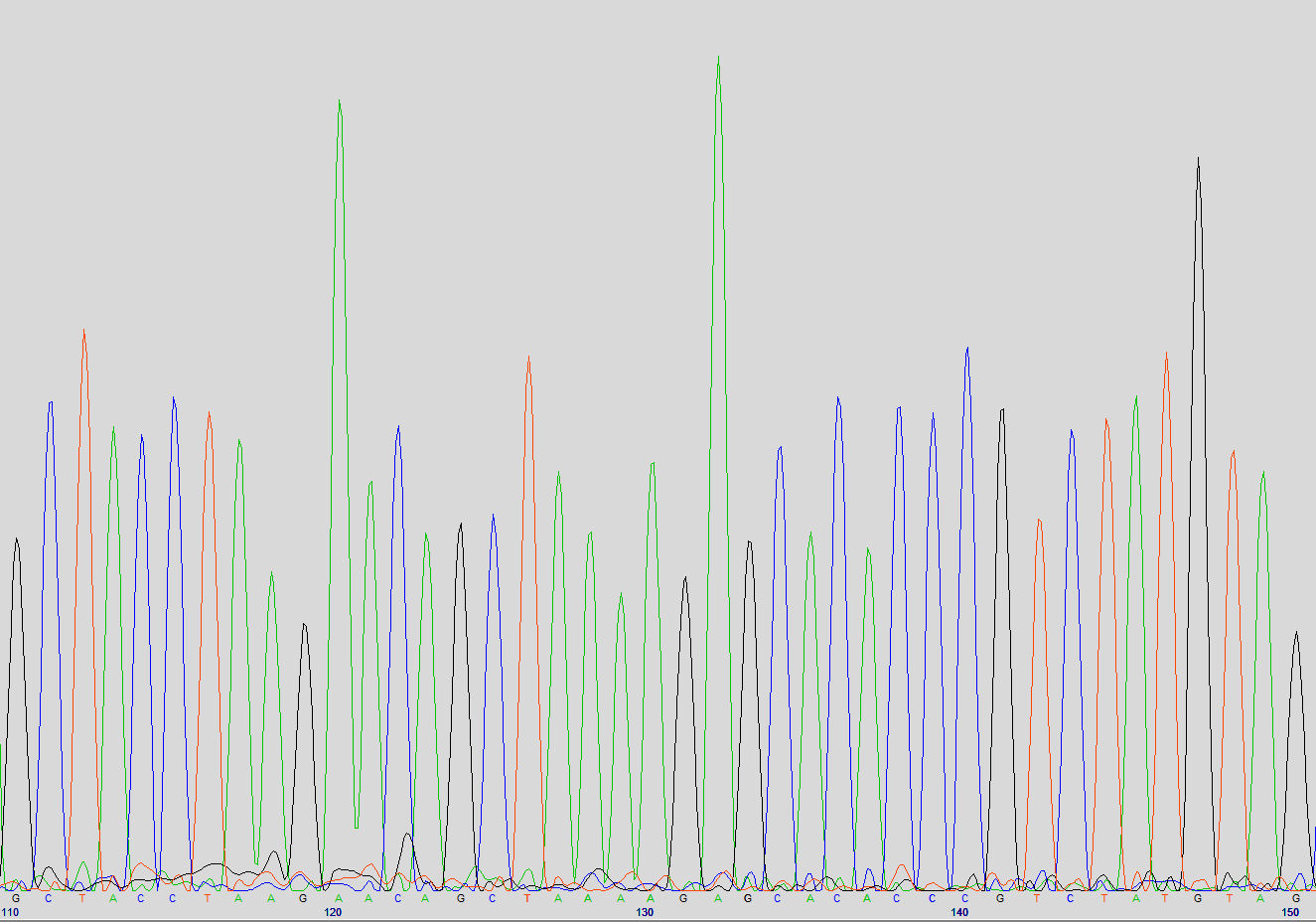


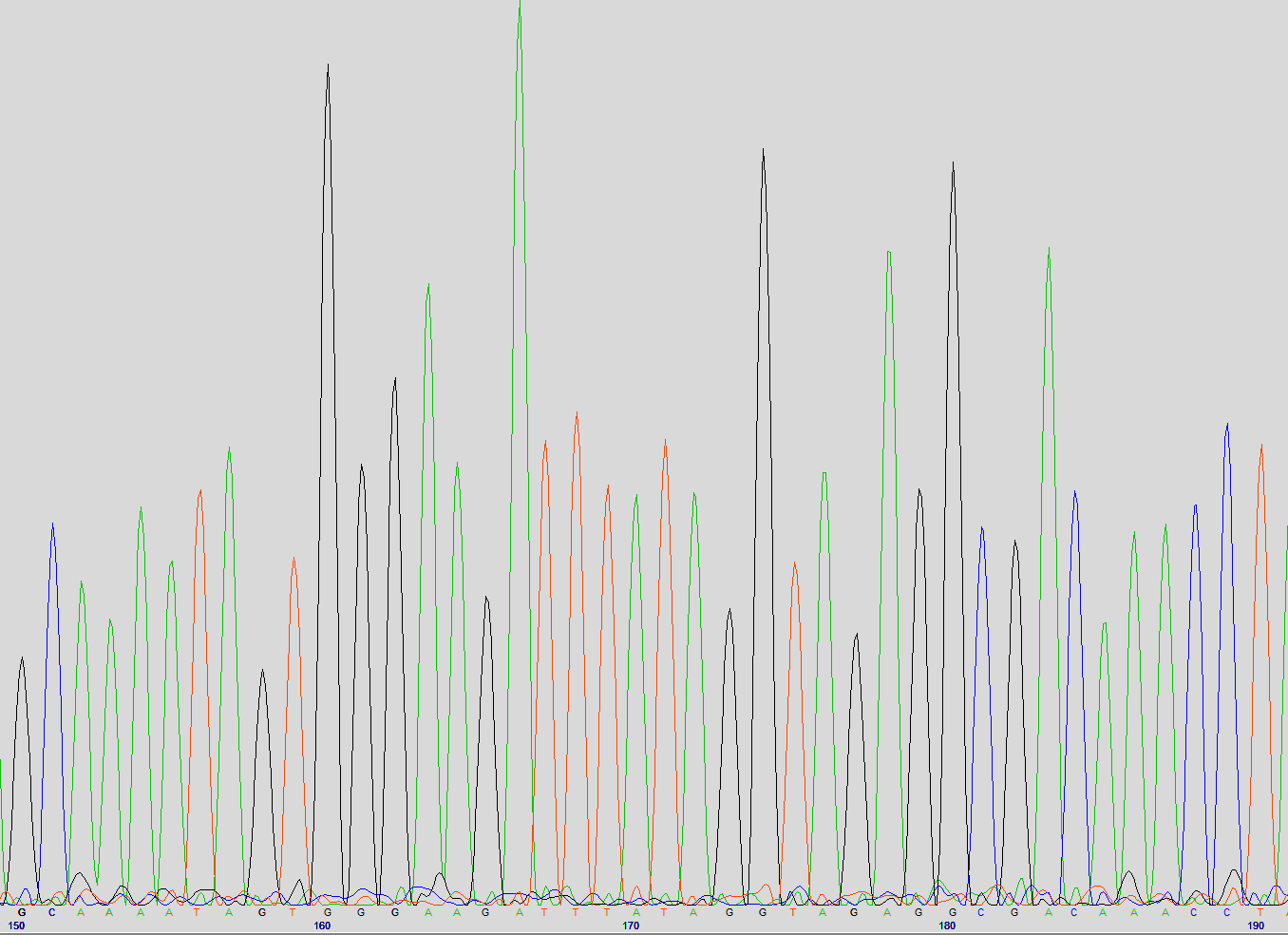


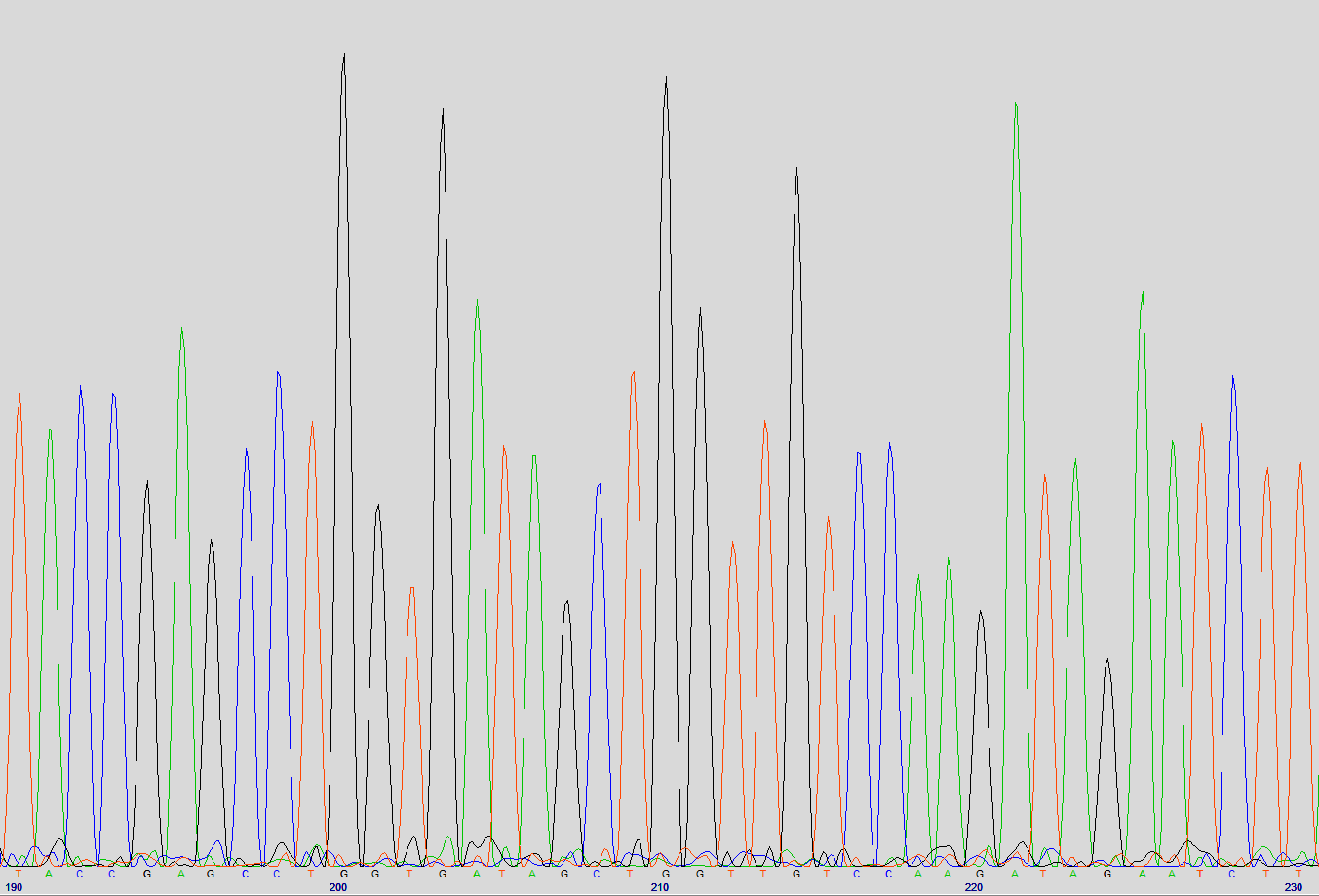


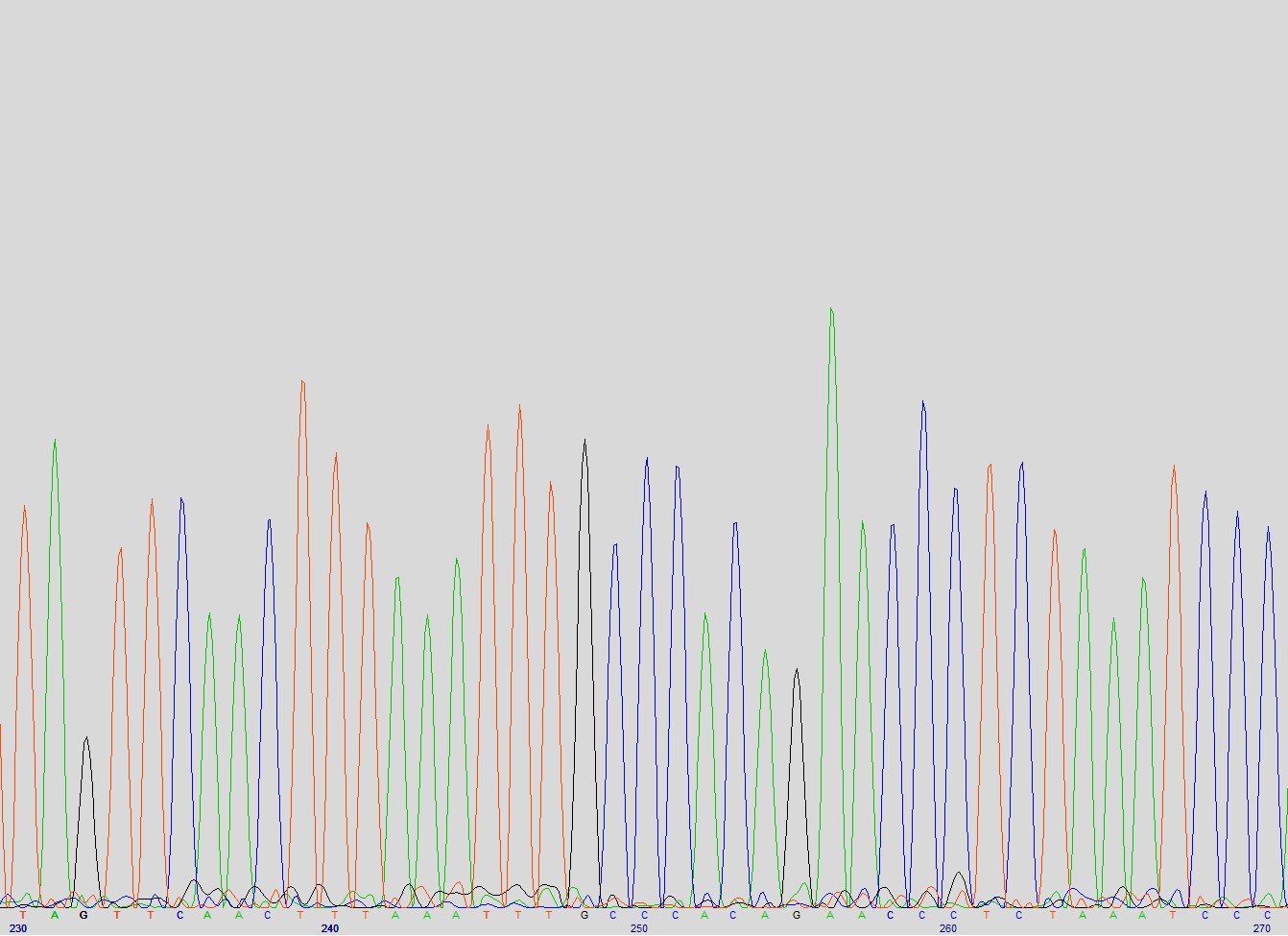


**Figure S-1: Human BLAST analysis of the exosomal mtDNA clones.** PCR product was obtained using 16S ribosomal RNA primers that amplify region 1873-2078 (Homo sapiens mitochondrion, complete genome NC_012920.1). The amplification product was ligated with Invitrogen™ pCR4TOPO-TA sequencing vector. The clones were selected on Ampicillin and Sanger sequenced by GENEWIZ®. The sequences are analyzed using the Basic Local Alignment Search Tool (BLAST). SNPs are highlighted by a red rectangle in the BLAST analysis and with an arrow in the chromatograms. **[A]** NSC exosomes: SNP @ 1893 A > -. **[B]** GBM exosomes: Deletion/SNP @ 1884 C>-. **[C]** iPS-NSC and iPS-NSC-AD have 100% sequence identity with each other as well as with mitochondrial genomic sequence, and no SNP. The chromatogram depicts the sequence of iPS-NSC, as an example.
