# Supplementary material for: Exposure to a Pathological Condition May Be Required for the Cells to Secrete Exosomes Containing mtDNA Aberration
Source: J Nucleic Acids. 2022 Mar 17;2022:7960198. doi: 10.1155/2022/7960198 (PMC9020996; doi:10.1155/2022/7960198)
Supplement: Supplementary materials — Figure S-1: human BLAST analysis of the exosomal mtDNA clones. PCR product was obtained using 16S ribosomal RNA primers that amplify region 1873-2078 (Homo sapiens mitochondrion, complete genome NC_012920.1). The amplification product was ligated with Invitrogen™ pCR4TOPO-TA sequencing vector. The clones were selected on ampicillin and Sanger sequenced by GENEWIZ®. The sequences are analyzed using the Basic Local Alignment Search Tool (BLAST). SNPs are highlighted by a red rectangle. (A) NSC exosomes: SNP @ 1893 A>-. (B) GBM exosomes: deletion/SNP @ 1884 C>-. (C) iPS-NSC and iPS-NSC-AD have 100% sequence identity with each other as well as with mitochondrial genomic sequence and no SNP. Figure S-2: human BLAST analysis of the exosomal mtDNA clones. PCR product obtained using tRNA-Leu (UUR) primers that amplify region 3212-3319 (Homo sapiens mitochondrion, complete genome NC_012920.1). The amplification product was ligated with Invitrogen™ pCR4TOPO-TA sequencing vector. The clones were selected on ampicillin and Sanger sequenced by GENEWIZ®. The sequences are analyzed using the Basic Local Alignment Search Tool (BLAST). NSC, GBM, iPS-NSC, and iPS-NSC-AD exosomal clones share 100% identity with each other as well as with mitochondrial genomic sequence. Figure S-3: human BLAST analysis of the exosomal mtDNA clones. PCR product obtained using NADH dehydrogenase subunit 1 primers that amplify region 3458-3561 (Homo sapiens mitochondrion, complete genome NC_012920.1). The amplification product was ligated with Invitrogen™ pCR4TOPO-TA sequencing vector. The clones were selected on ampicillin and Sanger sequenced by GENEWIZ®. The sequences are analyzed using the Basic Local Alignment Search Tool (BLAST). SNPs are highlighted by a red rectangle. (A) NSC exosomes: SNP @ 3502, T>A. (B) iPS-NSC exosomes: SNP @ 3545 C>-. (C) GBM and iPS-NSC-AD exosomes have 100% sequence identity with each other as well as with mitochondrial genomic sequence and no SNP. Figure S-4: GBM exosome D [file 7960198.f1.zip › S-4_rev2.docx]

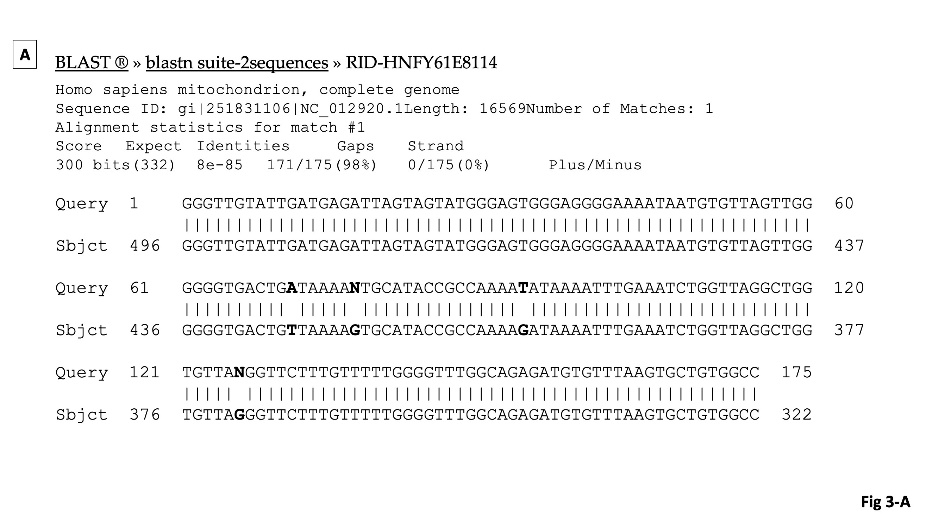


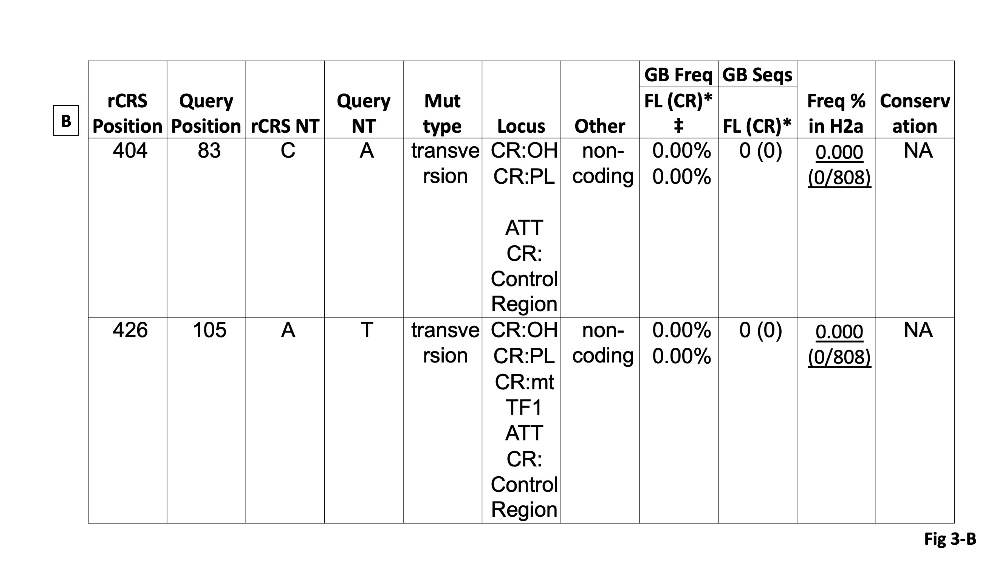


**C**


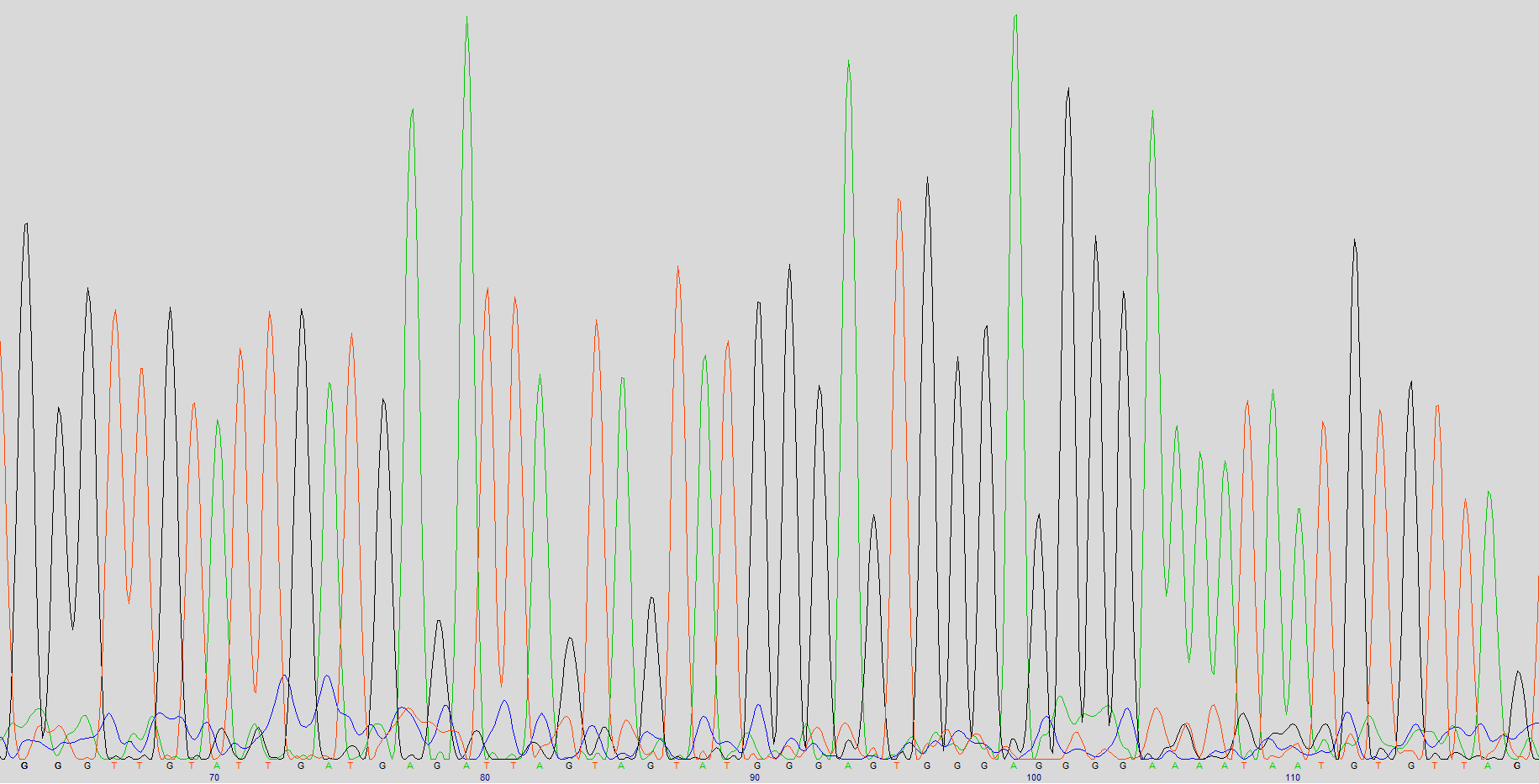


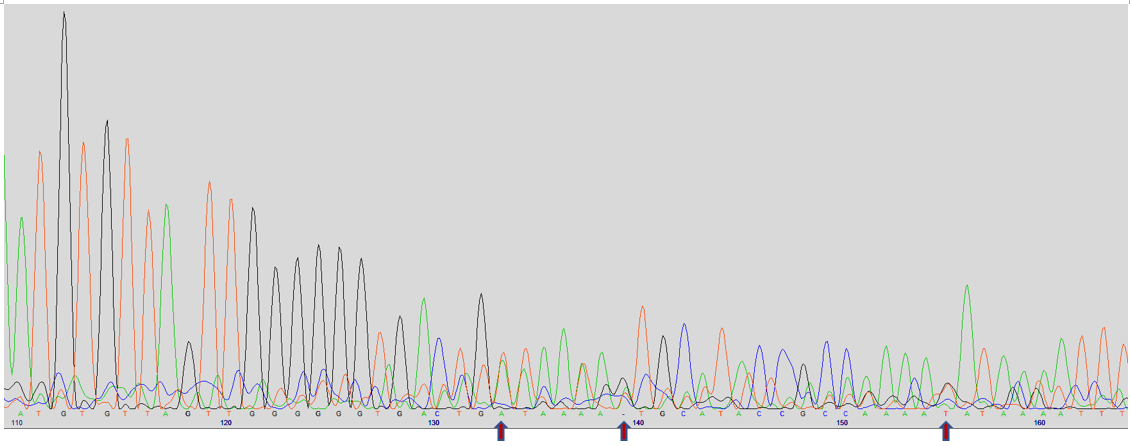


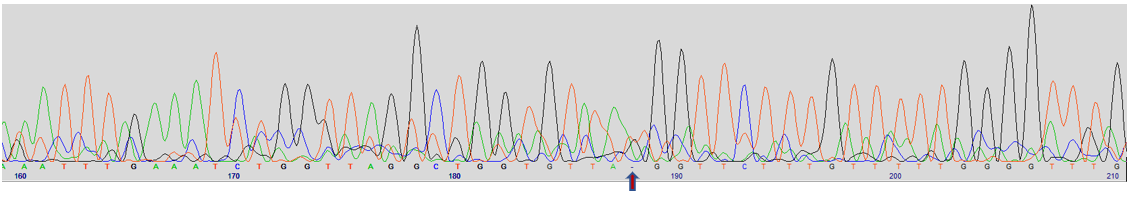


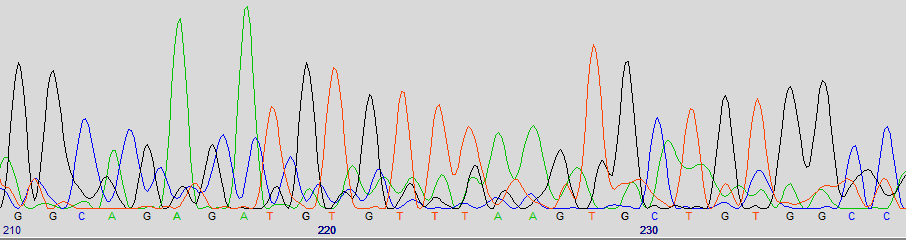


**Figure S-4:** GBM exosomes D-loop clone-1. (A) BLAST analysis against Homo sapiens mitochondrion, complete genome NC_012920.1: CD63+ exosomal mtDNA was amplified with D-loop primers (location: 321-496). PCR products were cloned into the pCR4-TOPO-TA vector. SNPs at 371 G > N, 426 T > A, 420 G > N, 404 G > T. (B) rCRS position according to MITOMAP: at 404: C > A, at 426: A >T, mutation type transversion. Information from Mitomap: * The current GB frequency data is derived from two sets of human mitochondrial sequences from GenBank: 52,633 full-lengths (FL) sequences (>15.4 kbp) and 74,970 short, control region (CR) containing sequences (0.4-1.6 kbp). ‡ High-Frequency Haplogroups: Variants found in haplogroups at 50% or higher are marked with a flag. (C) The chromatogram depicts the sequence of the entire PCR product cloned. The red arrows correspond to the SNPs.
