# Supplementary material for: Exposure to a Pathological Condition May Be Required for the Cells to Secrete Exosomes Containing mtDNA Aberration
Source: J Nucleic Acids. 2022 Mar 17;2022:7960198. doi: 10.1155/2022/7960198 (PMC9020996; doi:10.1155/2022/7960198)
Supplement: Supplementary materials — Figure S-1: human BLAST analysis of the exosomal mtDNA clones. PCR product was obtained using 16S ribosomal RNA primers that amplify region 1873-2078 (Homo sapiens mitochondrion, complete genome NC_012920.1). The amplification product was ligated with Invitrogen™ pCR4TOPO-TA sequencing vector. The clones were selected on ampicillin and Sanger sequenced by GENEWIZ®. The sequences are analyzed using the Basic Local Alignment Search Tool (BLAST). SNPs are highlighted by a red rectangle. (A) NSC exosomes: SNP @ 1893 A>-. (B) GBM exosomes: deletion/SNP @ 1884 C>-. (C) iPS-NSC and iPS-NSC-AD have 100% sequence identity with each other as well as with mitochondrial genomic sequence and no SNP. Figure S-2: human BLAST analysis of the exosomal mtDNA clones. PCR product obtained using tRNA-Leu (UUR) primers that amplify region 3212-3319 (Homo sapiens mitochondrion, complete genome NC_012920.1). The amplification product was ligated with Invitrogen™ pCR4TOPO-TA sequencing vector. The clones were selected on ampicillin and Sanger sequenced by GENEWIZ®. The sequences are analyzed using the Basic Local Alignment Search Tool (BLAST). NSC, GBM, iPS-NSC, and iPS-NSC-AD exosomal clones share 100% identity with each other as well as with mitochondrial genomic sequence. Figure S-3: human BLAST analysis of the exosomal mtDNA clones. PCR product obtained using NADH dehydrogenase subunit 1 primers that amplify region 3458-3561 (Homo sapiens mitochondrion, complete genome NC_012920.1). The amplification product was ligated with Invitrogen™ pCR4TOPO-TA sequencing vector. The clones were selected on ampicillin and Sanger sequenced by GENEWIZ®. The sequences are analyzed using the Basic Local Alignment Search Tool (BLAST). SNPs are highlighted by a red rectangle. (A) NSC exosomes: SNP @ 3502, T>A. (B) iPS-NSC exosomes: SNP @ 3545 C>-. (C) GBM and iPS-NSC-AD exosomes have 100% sequence identity with each other as well as with mitochondrial genomic sequence and no SNP. Figure S-4: GBM exosome D [file 7960198.f1.zip › S-7_rev2.docx]

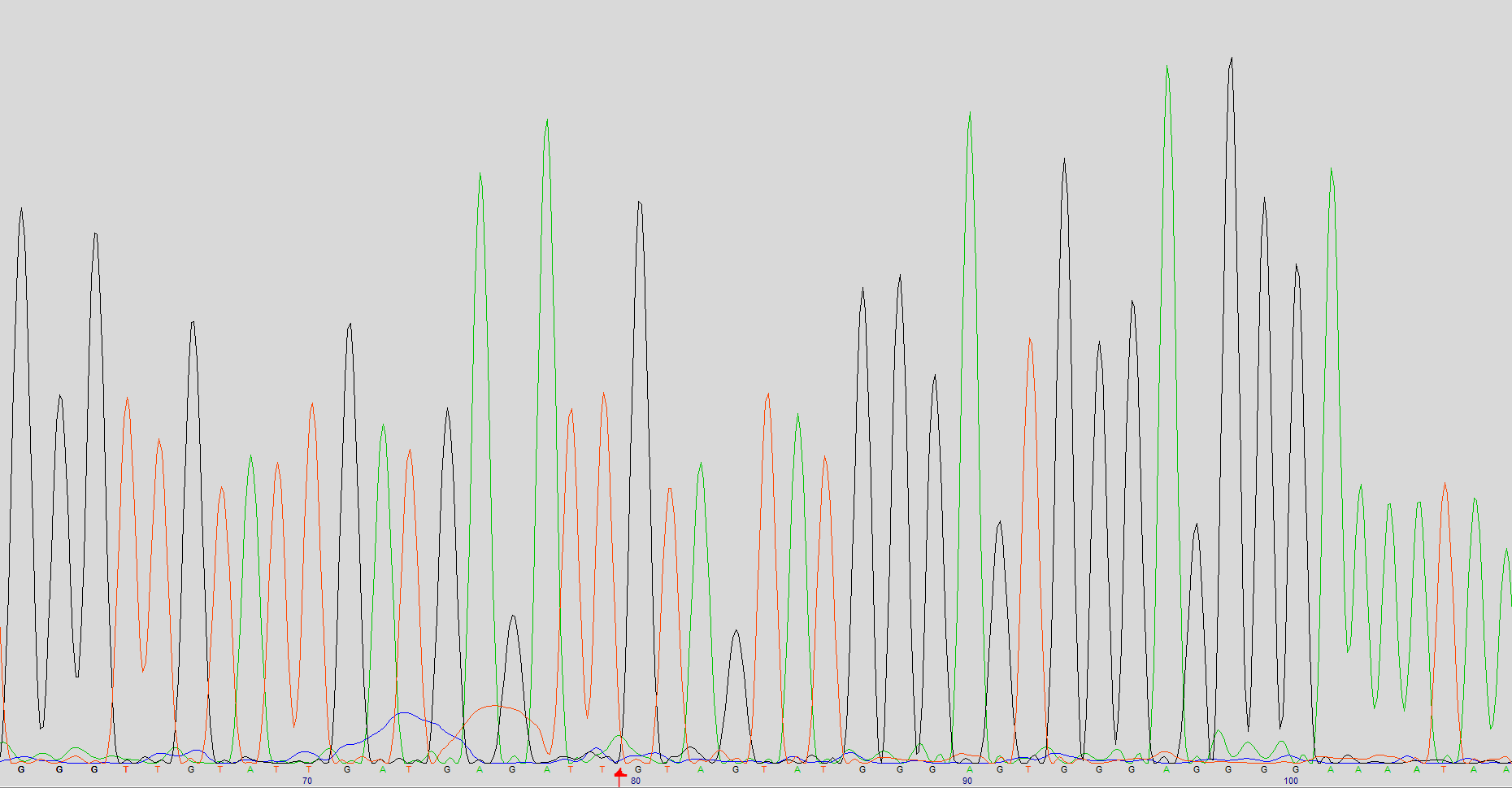


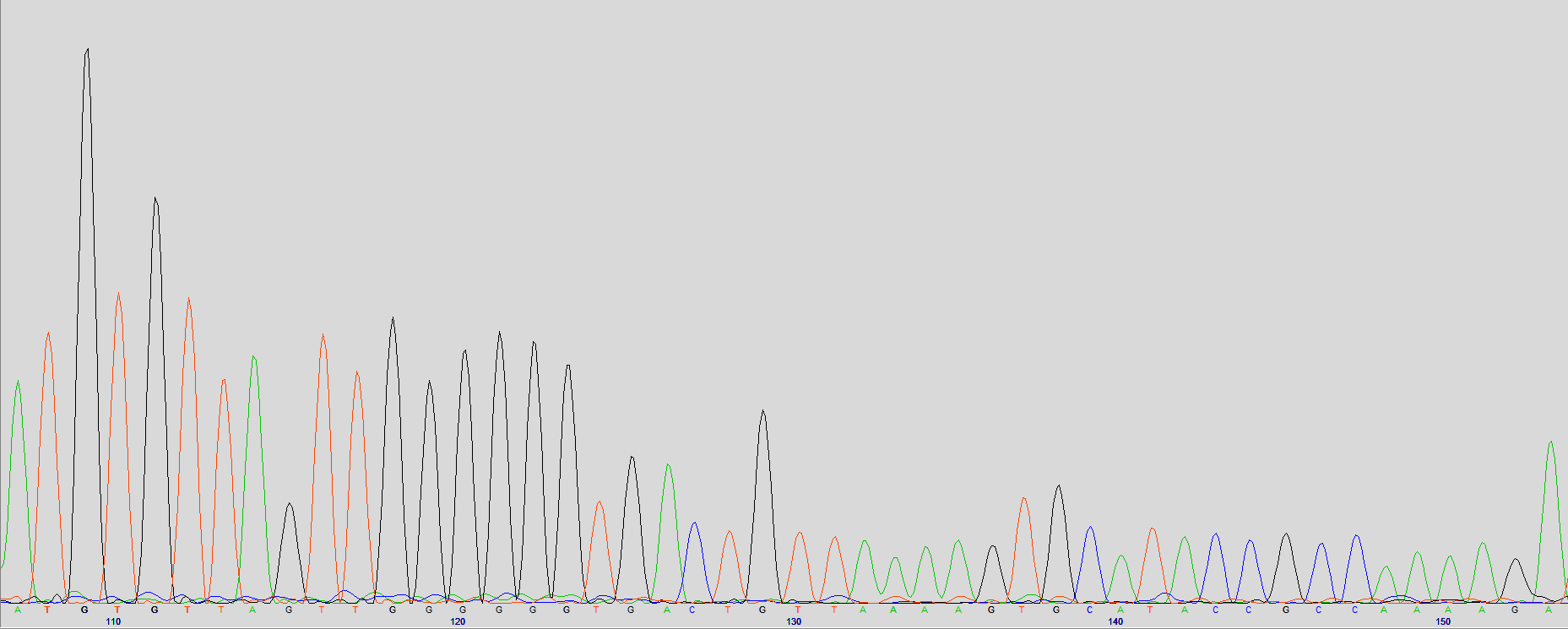


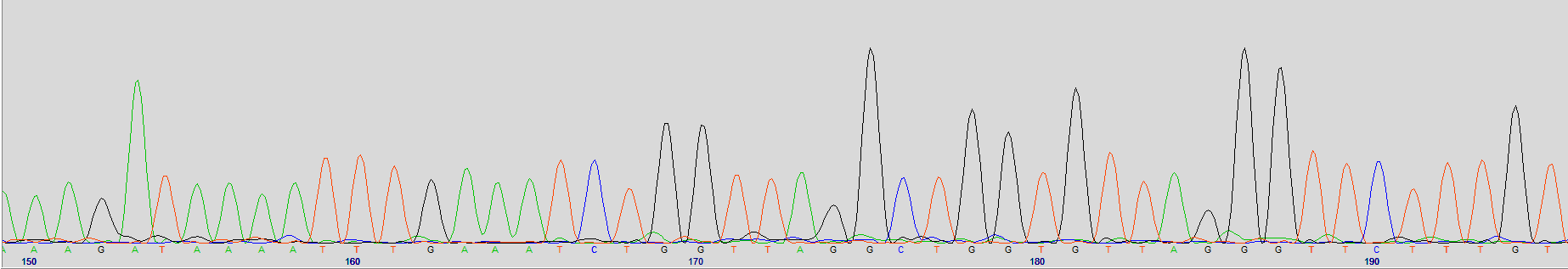


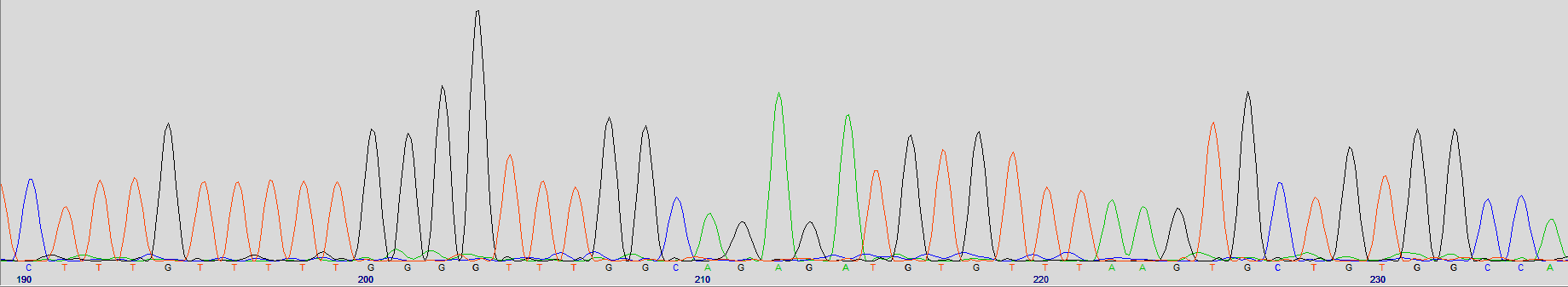


**Figure S-7: Human BLAST analysis of the exosomal mtDNA NSC exosomes D-loop clone. A detailed chromatogram for Figure 3B.** PCR product was obtained using D-loop RNA primers that amplify region 321-496 (Homo sapiens mitochondrion, complete genome NC_012920.1). The amplification product was ligated with Invitrogen™ pCR4TOPO-TA sequencing vector. The clones were selected on Ampicillin and Sanger sequenced by GENEWIZ®. The sequences are analyzed using the Basic Local Alignment Search Tool (BLAST). The red arrow in the sequence figure indicates the position of the nucleotide deletion in exosomal DNA in Homo sapiens mitochondrion complete genome sequence (NC_012920.1).
